# Using the Health Belief Model to Predict the Uptake of Mammographic Screening Among Saudi Women

**DOI:** 10.7759/cureus.11121

**Published:** 2020-10-24

**Authors:** Rana AlJunidel, Maram Alaqel, Sara H AlQahtani, Areeb M AlOgaiel, Faisal ALJammaz, Sulaiman Alshammari

**Affiliations:** 1 Medicine and Surgery, King Khalid University Hospital, Riyadh, SAU; 2 Family and Community Medicine, Colleage of Medicine, King Saud University, Riyadh, SAU

**Keywords:** breast cancer, health belief model, mammogram, saudi arabia

## Abstract

Background

Breast cancer (BC) is known to be the second leading cause of cancer deaths after lung cancer in Saudi Arabia. Early detection using screening methods can improve disease outcomes. In Saudi Arabia, the rates of BC screening are very low although it is a country that provides free healthcare services. This discrepancy between the availability of free healthcare services and women not utilizing these services necessitates an in-depth understanding of the health-related beliefs and barriers towards BC screening among Saudi women.

Objective

We aimed to use the Health Belief Model (HBM) to predict the uptake of mammographic screening among Saudi women. The secondary objective was to determine the knowledge, attitudes, and practices related to BC and mammography screening.

Research methodology

This was an analytical cross-sectional study using a self-administered questionnaire, which consisted of sociodemographic data, family history of BC, general information about BC, and the Champion’s Health Belief Model Scale (CHBMS). The study was conducted among Saudi women visiting the King Khalid University Hospital in Riyadh from September 2018 to February 2019. The data were analyzed using SPSS Statistics software version 26.00 (IBM, Armonk, NY).

Results

A total of 401 females participated in the study; the median age of the subjects was 49 years [interquartile range (IQR): 43-53 years]. Among them, 69.6% were married and 21.1% had a family history of BC. There was a statistically significant association of younger age, marital status, and a positive family history of BC with women undergoing mammographic screening (p<0.05).

Of the participants, 61.6% knew about the warning signs of BC, while only 59.9% were aware of the risk factors associated with it. The binary logistic regression did not show any significant association between CHBMS and mammogram screening. We concluded that the CHBMS components cannot be used in isolation to predict the risk of not undergoing mammogram screening. However, barriers and motivation components along with the knowledge and other factors can be used to predict mammogram screening.

Conclusion

Among our cohort of Saudi women, 62.1% had general awareness about BC, and younger age, marital status, and positive family history of BC were significantly associated with women undergoing mammography screening. The CHBMS components cannot be used in isolation to predict the risk of not undergoing mammogram screening, while barriers and motivation components along with the knowledge and other factors can be used to predict mammogram screening.

## Introduction

Breast cancer (BC) is known to be the most common cancer among women worldwide [1]. In 2015, it was found to be the second leading cause of cancer deaths after lung cancer in Saudi Arabia [2,3]. In the last 24 years, the incidence rates of BC in Arab women have increased; however, they are still often diagnosed at more advanced stages and at an earlier age when compared to women in western countries [3]. In Saudi Arabia, female BC was the most common cancer among Saudi women for 14 consecutive years, from 1994-2007, as per the report of the Saudi Cancer Registry [1]. With the growth of the Saudi population and aging among people, it is expected that an increase in BC incidence would occur over the coming decades [4]. Early diagnosis is critical and plays a significant role in cancer control, and delay in diagnosis leads to poor survival in BC patients [5]. Several studies have reported that BC mortality can be reduced by 23% using mammographic screening [3]. Despite this evidence, low participation rates in screening activities have been consistently reported among Arab women [3], and rates of BC screening are very low in Saudi Arabia although it is a country that provides free healthcare services [4]. In 2015, a very high noncompliance rate related to BC screening measures (89%) was reported in Saudi Arabia [6]. This discrepancy between the availability of free healthcare services and women not utilizing these services necessitates an in-depth understanding of the health-related beliefs and barriers toward BC screening among Saudi women. It is crucial to assess and identify factors that influence patient delay, in order to introduce new strategies and take immediate actions to shorten this delay and to improve medical help-seeking behavior among Saudi women to ensure early diagnosis and a better disease outcome [5].

Since studies related to knowledge, attitudes, and practices around BC in the Kingdom of Saudi Arabia have been scarce, and given the expected increase in the incidence of BC in the next decades in our nation, it is crucial to assess and identify factors that cause poor screening practices among patients, as it would help introduce new strategies and take immediate actions to ensure early diagnosis and a better disease outcome.

## Materials and methods

We conducted this cross-sectional study in the primary healthcare clinics at the King Khalid University Hospital (KKUH) from September 2018 to February 2019. The study included Saudi women aged between 40-69 years who were eligible for mammography screening and had not been diagnosed with BC. All women of this age range were eligible to participate in the study. All women who were not in the age range of 40-69 years and those who were diagnosed with BC previously were excluded.

To arrive at the sample size, we used the formula of a single proportion N = Zα² P(1-P)/d² where:

N = sample size

Zα = 1.96 for 95% confidence level 

P = 58.2% (7)

D = (5%) 

The calculated sample size for the present study was 374. To account for missing data, we included 401 participants. All women meeting the criteria were allowed to participate in the study.

Data collection was done using a self-administered questionnaire, which consisted of the following sections: sociodemographic characteristics, family history of BC, general information about BC, and Champion’s Health Belief Model Scale (CHBMS).

Sociodemographic factors recorded included marital status, occupation, and educational level. Regarding general knowledge, questions were sub-grouped into general information about BC, warning signs, and risk factors. The possible answers included either yes or no. We gave 1 point for correct answers and 0 for wrong answers. With regard to CHBMS, it consisted of the following five variables: susceptibility, seriousness, benefits, barriers, and health motivation. The items under each variable were scored according to the Likert scale as follows: strongly disagree = 1, strongly agree = 5.

We summed up each participant's score, and then took the median and interquartile range (IQR) for each variable; the parameters were used to compare the participants’ screening practices.

Ethical considerations

The study was approved by the Institutional Review Board (IRB) at King Saud University (project number E-18-3301). All participant-related information was kept anonymous, and participants' privacy and confidentiality were ensured. The authors had no conflict of interest. Written consent was signed by all the participants after we explained the nature and purpose of the study.

Statistical analysis

SPSS Statistics version 26.00 (IBM, Armonk, NY) was used to analyze the data. All the numerical variables were not normally distributed. Thus, the median (Mdn) and IQR were used to summarize the numerical data along with the proportion for categorical variables. Mann-Whitney U and Kruskal-Wallis tests were used to compare the means. The chi-squared test and logistic regression analysis were used to find the significance for categorical outcomes. Prior to performing the regression analysis, bivariate analysis was done to identify the variables significantly associated with the outcome to be enrolled for the regression model. As for the components of the Health Belief Model (HBM), binary logistic regression was done only for the CHBMS components in a separate model to meet the objective of the current study.

## Results

A total of 401 women participated in the study. The median age of the subjects was 49 years (IQR: 43-53). The majority (65.8%) were aged 50 years or less; 69.6% were married and 21.1% were aware of having a family history of BC (Table 1). Only 41% of the participants had undergone mammography before. There was a statistically significant association of younger age and positive family history of BC with undergoing mammographic screening (p<0.05). 

Correct answers were given for 61.6% of the questions related to warning signs, while 59.9% of the participants answered risk factors questions. Moreover, 62.1% answered general knowledge questions correctly (Table 2). Also, regarding Saudi women’s knowledge of BC mammography screening, 45.5% of the subjects knew about the clinical examination of breasts and 89.9% knew that it could enhance the chances of recovery. However, the knowledge about mammograms and breast self-examination (BSE) was inadequate (42.9% and 57.1% respectively).

**Table 1:** Sociodemographic characteristics and family history of breast cancer IQR: interquartile range

Variables	Number of participants	Percentage
Age in years (median: 49; IQR: 43–53)		
40–49	264	65.8
50–59	82	20.4
60–69	55	13.7
Marital status		
Single	31	7.8
Married	281	70
Separated	89	22.2
Employment status		
Employed	143	35.7
Not employed	258	64.3
Educational level		
Illiterate	38	9.4
Elementary	55	13.7
Secondary	132	32.9
Higher education	176	44
Family history of breast cancer		
Yes	83	20.7
No	318	79.3

**Table 2:** Correct answers for Saudi women’s knowledge about breast cancer mammography screening

General knowledge about breast cancer and Saudi women's practices regarding mammography screening		
	Correct answers	
	Number	Percentage
General Knowledge about breast cancer		
Breast cancer is curable in the early stages?	335	83.5
Breast cancer is highly fatal without treatment?	294	73.3
Breast cancer is painless in the early stages?	229	57.1
Breast cancer is more common in women over 50?	257	64.1
Breast cancer occurs in one breast only?	202	50.4
Breast cancer is more common in obese women?	152	37.9
Nipple discharge is important?	266	66.3
A lump is definitely cancerous?	229	57.1
Knowledge domain of breast cancer warning signs		
Breast lump?	311	77.6
Early menarche?	111	27.7
Sudden and abnormal changes in size?	318	79.3
Discharges from nipples?	301	75.1
Changes in nipple shape?	308	76.8
Knowledge domain of breast cancer risk factors		
Radiotherapy?	183	45.6
Hormonal replacement therapy?	159	39.7
Obesity?	157	39.2
Practice physical exercise?	288	71.8
Smoking?	263	65.6
Alcohol?	251	62.6
Increase with age?	219	54.6
Low fat intake?	198	49.4
Late menopause?	104	25.9
Long intake of oral contraceptive pills?	234	58.4
Family history of breast cancer?	308	76.8
Breastfeeding practice?	318	79.3
Trauma to breast area?	100	24.9
Saudi women’s knowledge and practices regarding breast cancer screening	Yes	
	Number	Percentage
Did you do a mammogram before?	161	40.6
Do you know about mammograms?	170	42.9
Do you know how to perform breast self-examination?	226	57.1
Do you know about the clinical examination of breasts?	180	45.5
Is it possible for screening measures to enhance the chance of recovery?	358	89.9

We found that those who had already undergone mammograms had a higher mean rank of correct answers compared to those who had not, except for the barriers where those who had not undergone mammograms had a higher mean (204.35). Additionally, there was a statistically significant association between undergoing mammograms and the perception of susceptibility toward BC as well as the components of benefit, barriers, and health motivation (p<0.05) (Table 3).

Addressing the factors that affect mammogram screening in our region, we found that older females tended to undergo mammogram at a higher rate compared to those who were younger in age [odds ratio (OR): 1.069, 95% CI: 1.035-1.106]; females with a family history of BC tended to have a lower risk of not undergoing mammogram (OR: 0.523, 95% CI: 0.304-0.901). Level of education and employment were not found to significantly affect the rates of mammogram screening (p>0.05). A lack of knowledge correlated with the highest risk (OR: 1.256, 95% CI: 1.110-1.420). Lastly, the presence of barriers carried a significant risk for women not undergoing mammograms compared to those who did (OR: 0.905, 95% CI: 0.837-0.978) (Table 4).

We used the components of CHBMS to predict mammogram screening among Saudi women. The binary logistic regression did not show any significant association between CHBMS and mammogram screening. We concluded that the components cannot be used in isolation to predict the risk for mammogram screening (Table 5). However, barriers and motivation components along with the knowledge and other factors can be used, as shown in Table 4.

**Table 3:** Mann-Whitney mean scores for overall subscales of knowledge, susceptibility, seriousness, benefits, barriers, and health motivation related to undergoing mammograms

	Screening: did you undergo mammogram screening before?	Mean rank	P-value
Knowledge score	No	187.24	0.01
	Yes	217.55	
Seriousness score	No	186.50	0.327
	Yes	197.78	
Benefits score	No	178.97	0.001
	Yes	218.68	
Barriers score	No	204.35	0.026
	Yes	178.67	
Motivation score	No	179.44	0.001
	Yes	219.09	

**Table 4:** Logistic regression for factors affecting Saudi women undergoing mammogram screening

	Coefficient (B)	Odds ratio (95% CI)	P-value
Age	0.067	1.069 (1.035–1.106)	<0.001
Marital status	Single (-0.609); married (0.739); separated (reference)	0.544 (0.173–1.179)	0.297
Family history	-0.647	0.523 (0.304–0.901)	0.019
Barriers score	-0.100	0.905 (0.837–0.978)	0.012
Motivation score	0.084	1.088 (1.027–1.153)	0.004
Knowledge	0.228	1.256 (1.110–1.420)	<0.001

**Table 5:** Binary logistic regression for the Health Belief Model as a predictor for Saudi women undergoing mammography screening

	Did you do mammogram screening before?	
	Odds ratio (95% CI)	P-value
Seriousness score	1.026 (0.982–1.071)	0.251
Benefits score	1.058 (0.996–1.124)	0.068
Barriers sore	0.931 (0.863–1.003)	0.060
Motivation score	1.056 (0.998–1.117)	0.057

## Discussion

The median age of our participants was 49 years (IQR: 43-53). Most of them (65.8%) were aged 50 years or less; 69.6% were married and 21.1% were aware of having a family history of BC. In a study conducted in Turkey in 2019, the mean age of the participants was 36.1 ± 0.53 years (n = 200); most of them were between 30-40 years (51.5%). A majority of the subjects were married (57%), and only 7% were aware of having a positive family history among first-degree relatives [7].

**Figure 1:**
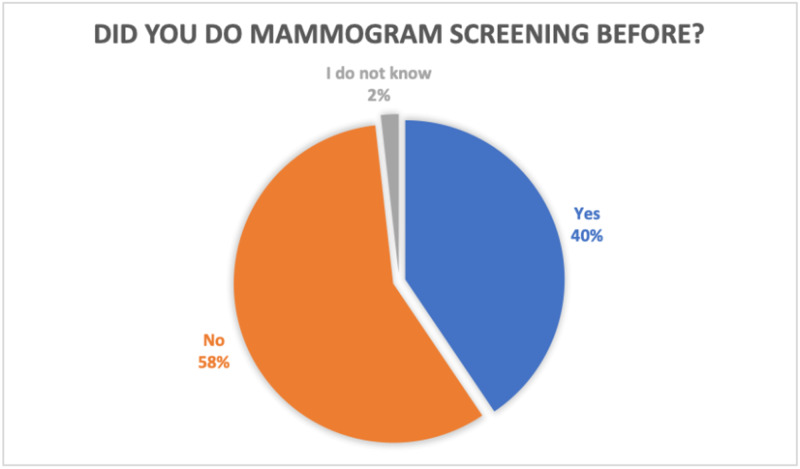
Graph showing the proportion of women who have done mammogram before

As shown in Figure 1, only 40% of the participants in our cohort had undergone mammograms previously. There was a statistically significant association of younger age, married status, and a positive family history of BC with women undergoing mammographic screening (p<0.05).

Our study showed that 40% of women had undergone mammogram screening previously. In a study conducted in Jordan in 2015, which had aimed to explore the knowledge, barriers, and attitudes regarding BC mammography screening, it was concluded that in the absence of regular, systematic screening for BC in Jordan, the uptake of this preventive service was very low [8]. In 2015, a study was done in Saudi Arabia with the aim to explore the perception towards BC and BSE among Saudi women using the HBM. It concluded that Saudi women had poor knowledge about BC, along with poor BSE practices and a negative attitude towards BSE [9].

Another study conducted in Malaysia in 2011 aimed to assess the role of awareness and the practice of BSE in detecting breast abnormalities. Many of the study participants (57.9%) were unaware that they were at risk of developing BC. Also, most of them were not performing BSE due to a lack of knowledge about how to do it [10]. Another study in 2015 aimed to assess if the HBM can predict BC screening behaviors. The study showed the need for establishing educational programs that should focus on imparting the skills related to BSE and enlightening women about the benefits of healthy behaviors and eliminating barriers that affected them [11]. However, among our study subjects, 62.1% showed a general knowledge about BC and 61.6% knew about the warning signs. However, fewer women were able to recognize the predisposing risk factors, which was not ideal since merely knowing about the warning signs would not help in preventing the disease from the beginning. Additionally, 45.5% of the subjects knew about clinical examination of breasts, and 89.9% knew that it could enhance the chances of recovery. However, the knowledge about mammograms and BSE was limited (42.9% and 57.1% respectively). 

Mammographic screening is considered to be one of the vital components in the early diagnosis of BC. However, the majority of Saudi women do not undergo it although it is a free healthcare service in Saudi Arabia [4]. Also, a cross-sectional study conducted in Riyadh, Saudi Arabia in 2016 indicated that help-seeking behaviors among the community required improvement as 37% of the participants relied on self-help or tended to consult relatives [12]. Our results showed that the younger age group (<50 years old) tended to perform fewer mammograms compared to other age groups. This finding could be attributed to their educational level and ability to use technology and social media [13]. Also, those who had a positive family history demonstrated higher screening rates. Moreover, based on the HBM mean score, all parameters were higher in those who underwent a mammogram compared to those who did not, except for the barriers parameter, which included fear of disease, shyness, fear of pain, concerns about the cost, and concerns about the long duration of the procedure. Those who had not undergone a mammogram scored 204.35 compared to 178.67 in those who had. This indicates the presence of barriers and misconceptions that should be eliminated. Moreover, the barriers carry significant risk factors for women not undergoing mammograms (OR: 0.905, p: 0.012). This finding is consistent with a study done in Turkey in 2019, which concluded that females who underwent mammography had lower barriers to mammography compared to females who did not [7].

## Conclusions

Based on our findings, 62.1% of our cohort of Saudi women had general awareness about BC, and younger age, marital status, and a positive family history of BC were significantly associated with mammographic screening among Saudi women. We concluded that the CHBMS components cannot be used in isolation to predict the risk of not undergoing mammogram screening. However, barriers and motivation components along with the knowledge and other factors can indeed be used to predict mammogram screening among women.
